# Neurocognitive Outcome After Treatment With(out) ECMO for Neonatal Critical Respiratory or Cardiac Failure

**DOI:** 10.3389/fped.2019.00494

**Published:** 2019-11-26

**Authors:** Raisa M. Schiller, Dick Tibboel

**Affiliations:** Department of Pediatric Surgery/IC Children and Child and Adolescent Psychiatry/Psychology, Erasmus MC-Sophia Children's Hospital, Rotterdam, Netherlands

**Keywords:** ECMO (extracorporeal membrane oxygenation), Newborn, respiratory failure, circulatory failure, hippocampus

## Abstract

Over the years, it has become clear that children growing up after neonatal critical illness are at high risk of long-term neurocognitive deficits that impact their school performance and daily life activities. Although the pathophysiological mechanisms remain largely unknown, emerging evidence seems to suggest that long-term neuropsychological deficits following neonatal critical illness are not associated with the type of treatment, such as extracorporeal membrane oxygenation (ECMO), but rather with underlying disease processes. In this review, neurocognitive outcome and brain pathology following neonatal critical respiratory and cardiac illness, either treated with or without ECMO, are described and compared in order to gain insight into potential underlying pathophysiological mechanisms. Putting these findings together, it becomes apparent that both children with complex congenital heart disease and children who survived severe respiratory failure are at risk of neurocognitive deficits later in life. Neurorehabilitation strategies, such as Cogmed working-memory training, are discussed. While prevention of neurocognitive deficits altogether should be strived for in the future, this is not realistic at this moment. It is therefore of great importance that children growing up after neonatal critical illness receive long-term care that includes psychoeducation and personalized practical tools that can be used to improve their daily life activities.

## Introduction

Extracorporeal membrane oxygenation (ECMO) can be used as a lifesaving therapy in critically ill neonates with severe refractory respiratory and/or cardiac failure. As more and more of these patients survive to discharge (73% following respiratory illness and 42% after cardiac illness) (1), long-term outcomes become increasingly important. It has become clear that children growing up after neonatal critical illness are at high risk of long-term neurocognitive deficits that have a profound impact on school performance and daily life activities (2). Survivors are at risk of ‘growing into deficit’ as subtle brain injuries acquired at a young age only become functionally evident over time when demands on cognitive functioning increases (3). This phenomenon is nested within different developmental processes that occur in the brain [e.g., myelination, synaptic pruning, and neurogenesis (4)] during the period of critical illness.

The pathophysiological mechanisms underlying the long-term neurodevelopmental deficits remain largely unknown. Most likely, a complex interplay amongst different factors associated with the underlying disease (pharmacological), treatment and “iatrogenesis,” further complicated by the child's genetic predisposition (5) and social economic status (6), determines a child's neurodevelopment. Emerging evidence seems to suggest that long-term neuropsychological deficits following neonatal critical illness are not associated with the type of treatment, such as extracorporeal membrane oxygenation (ECMO) (7), but rather with underlying disease processes, such as hypoxia-ischemia, stress, and neuroinflammation (2, 8, 9). However, whether this is similar between neonates with severe respiratory failure and neonates with cardiac anomalies remains largely unknown. Furthermore, whether brain alterations and its neurocognitive consequences are selective or generalized in these patients and differs between patients treated with and without ECMO is not yet clear.

In this review, long-term neurocognitive outcome and brain pathology following neonatal critical respiratory and cardiac illness, either in children treated with or without ECMO, will be described and compared. In light of this, potential common pathophysiological mechanisms across these patients will be explored. Finally, suggestions to improve long-term neurodevelopmental care, both through intervention and counseling, will be provided.

## Neurodevelopmental Outcome After Severe Respiratory Failure, Treated With or Without ECMO

There are relatively few studies that have compared outcome between ECMO and non-ECMO treated patients after severe respiratory failure (Table 1A). In one of the first studies comparing outcome between 7-year-old children treated with neonatal ECMO to children treated with conventional management (CM) in response to severe respiratory failure, global cognitive loss, poor spatial skills, difficulties with reading comprehension and deficits in visual and verbal memory were found equally in both groups (12). Madderom et al. (11) directly compared CDH survivors treated with (*n* = 16) or without ECMO (*n* = 19) on IQ, school performance and sustained attention at 8 years of age. Mean IQ significantly differed between the ECMO group [91.7 (19.5)] and non-ECMO group [111.6 (20.9)], but was normal in both groups compared to the general population. The proportions of children with above average, average and below average IQ did not differ significantly between both groups (*p* = 0.052), but there was a trend toward more children with below average IQ in the ECMO group compared to the non-ECMO group. In both groups, however, twice as many children needed extra help in school compared to healthy peers and both groups showed significantly impaired sustained attention compared to healthy peers (11). The difficulties experiences in school by these children therefore seem to be largely independent of IQ.

**Table 1:** Studies assessing neurocognitive outcome in ECMO vs. non-ECMO treated patients for severe respiratory failure or cardiac failure.

**References**	**Participants**	**Assessed**	**Methods and materials**	**Findings**
**(A) SEVERE RESPIRATORY FAILURE**				
Cooper et al. (10)	40 children treated for acute hypoxic respiratory failure: 27 treated with ECMO and 13 with conventional management. 64 healthy controls	8–15 years	Neuroimaging: Structural MRI 1.5 Tesla. Neurocognitive domains: intelligence, memory	Smaller left, right and bilateral hippocampal volume in patients. No difference in hippocampal volume between patients treated with ECMO and conventional treatment. The ECMO and CM subgroups differed consistently on Learning and Delayed Recognition, in both of which the ECMO subgroup scored below the CM subgroup
Leeuwen et al. (9)	65 survivors of severe respiratory failure: 35 treated with ECMO (CDH and other) and 30 treated with conventional management (CDH)	8 years	Neurocognitive domains: intelligence, attention, verbal and visuospatial memory, executive functioning, visuospatial processing	Patients had average intelligence (mean intelligence quotient ± SD, 95 ± 16), but significantly poorer sustained attention and memory than the norm population. ECMO-treated CDH patients had significantly lower mean IQ (84 ± 12) than other neonatal ECMO patients (94 ± 10) and CDH patients treated with conventional management (100 ± 20). Mean (SD) IQ for the ECMO
Madderom et al. (11)	35 survivors of congenital diaphragmatic hernia: 16 treated with neonatal ECMO and 19 with conventional management	8 years	Neurocognitive domains: Intelligence, concentration and attention	Mean (SD) IQ for the ECMO group was 91.7 (19.5) vs. 111.6 (20.9) for the non-ECMO group (*p* = 0.015). For all participants, problems with concentration (68%, *p* < 0.001) and with behavioral attention (33%, *p* = 0.021) occurred more frequently than in reference groups, with no difference between treatment groups
McNally et al. (12)	90 survivors of severe respiratory failure: 56 treated with neonatal ECMO and 34 with conventional management	7 years	Neurocognitive domains: Cognitive ability (verbal, nonverbal reasoning, and spatial abilities), number skills, spelling, word reading, reading comprehension and visual and verbal memory	76% recorded a cognitive level within the normal range. Learning problems were similar in the 2 groups, and there were notable difficulties with spatial and processing tasks.
Schiller et al. (13)	38 children with CDH and/or treated with neonatal ECMO. No controls	8–12 years	Neuroimaging: Structural MRI and DTI 3Tesla. Neurocognitive domains: intelligence, attention, verbal and visuospatial memory, executive functioning, visuospatial processing	Mean diffusivity (MD) in the left parahippocampal region of the cingulum (PHC) was negatively associated with visuospatial memory. MD in the left and right PHC were negatively associated with verbal memory. Bilateral hippocampal volume was positively associated with verbal memory. No differences between groups in the structure-function associations found
**(B) SEVERE CARDIAC FAILURE**				
Tindall et al. (14)	9 survivors of cardiac disease treated with ECMO compared to 13 controls matched for cardiac disease and age of surgery. 31 healthy controls	4–6 years	Neurocognitive domains: general cognitive ability, sustained attention, memory, spatial construction, verbal ability	Children treated with ECMO demonstrated significant impairment in general cognitive ability compared to normal controls. No group differences were found in impulsivity or sustained attention. On lateralized measures, children treated with ECMO demonstrated significant impairment in left-hand motor skill, visual memory, and spatial construction compared to both cardiac controls and normal controls. There were no group differences in general verbal ability, verbal memory, right-hand motor skills, or tactile perception

*ECMO, extracorporeal membrane oxygenation; CDH, congenital diaphragmatic hernia; CM, conventional management; MRI, magnetic resonance imaging; DTI, diffusion tensor imaging; MRS, magnetic resonance spectroscopy.*.

Indeed, when elaborate neuropsychological assessment was used to compare outcome between 8-year-old survivors of congenital diaphragmatic hernia (CDH) survivors treated with neonatal ECMO (CHD-ECMO), CDH survivors treated without ECMO (CDH-non-ECMO) and survivors of other types of severe respiratory failure treated with neonatal ECMO (ECMO-other), lower IQ in the CDH-ECMO group was found compared to the other groups. However, deficits in sustained attention and in verbal and visuospatial memory (both immediate and delayed recall) were found across all three groups, while other neuropsychological outcomes were normal (9). In all groups, the observed attention and memory problems were more severe than expected based on their IQ, indicating specific impairments in these domains that were independent of underlying diagnosis or ECMO treatment (9).

Behavioral problems have been compared between neonatal ECMO vs. non-ECMO treated survivors of respiratory failure as well. McNally et al. (12) found an increased risk of behavioral problems among 7-yearold children who had been treated conventionally, in particular hyperactivity. Madderom et al. compared self-perceived competence at 8 years of age and found no differences between groups. Furthermore, scores were similar to the general population. Behavioral problems did not differ between groups either, but the groups studied were small which makes the results less definitive (11). Future studies with large sample sizes are needed to gain a better understanding of how ECMO may or may not negatively influence behavioral outcome.

In recent years, studies have assessed long-term brain pathology and brain-function associations in these patient groups as well. Cooper et al. (10) assessed cognitive functioning and brain outcomes between 12-year-old survivors of acute hypoxemic respiratory failure (AHRF) who had either received treatment with neonatal ECMO or CM and were free of overt neurological impairment. In both groups, a similar degree of hippocampal atrophy was found at 12 years of age that positively correlated with memory outcome (10). The ECMO group was more impaired in Learning and Delayed Recognition relative to the CM subgroup, but their total IQ was lower as well (10). These findings thus seem to be similar to the results mentioned in the previous paragraph (9, 11), where specific neuropsychological deficits exist that are incongruent with their general intellectual abilities. Furthermore, the brain alterations seem to be specific as well. In another study, school-age survivors of neonatal ECMO and/or CDH, hippocampal volume reductions were observed, adjusted for total brain volume, that were associated with worse verbal memory delayed recall. This association was found both in children treated with and without ECMO (15). In the same cohort, white matter microstructure was assessed, showing alterations in the parahippocampal region of the cingulum, a white matter tract connecting the medial temporal lobe with the parietal and occipital lobes, to be associated with worse visuospatial memory (15). Again, these structure-function relationships existed in both the patients treated with and without ECMO (15).

Specific long-term neurodevelopmental deficits, particularly in memory and the hippocampus, seem to exist in survivors of critical illness, irrespective of ECMO treatment. Nonetheless, more widespread neuropsychological impairment has also been reported in survivors of neonatal ECMO compared to those treated with CM (9–12). These differences may be due to more global brain alterations as a result of greater illness severity in the neonatal period. In line with this, in the study by Cooper et al. (10) an increase in CSF and trend toward reduced global white matter was observed in the ECMO group compared to the CM group. On the contrary, although global alterations in white matter microstructure were found in survivors of severe neonatal respiratory failure by others as well, these were independent of ECMO treatment (13). The explanation of this discrepancy remains speculative, but may be because the differences are small and are therefore difficult to detect in relatively small study populations.

## Neurodevelopmental Outcome After Severe Cardiac Failure—Treated With or Without ECMO

In neonates with congenital or acquired heart disease, ECMO is used as a perioperative bridge to recovery or temporary support. Although respiratory failure remains the most common indication for extracorporeal life support today, the proportion of cardiac ECMO cases has increased dramatically. ECMO has evolved into a standard therapy for support of cardiac failure refractory to medical care alone (16). Fortunately, this has led to lower mortality rates in these patients and improvement of short-term outcome (1, 16). Because of this, monitoring long-term neurodevelopment in these patients is of great importance.

Several long-term studies have shown that neonates with congenital or acquired heart disease are at risk of long-term deficits in multiple neurocognitive domains, such as visuospatial skills, executive functioning, attention and memory (17–22). Subsequently, just as in patients with severe respiratory failure, survivors of cardiac failure within the first weeks of life are at increased risk of academic difficulties (17–21). Whether patients treated with ECMO are at an even higher risk of these long-term neurodevelopmental problems remains largely unknown. Patients in need of ECMO obviously represent a negative case selection due to circulatory failure post-cardiac repair. In some patients, such as in selected patients with TGA and pulmonary hypertension, survival is even dependent on pre-surgery support by ECMO.

Studies comparing neurocognitive outcome after severe cardiac failure between ECMO and non-ECMO treated patients are very scarce (Table 1B). In an early study on this topic, Tindall et al. (14) compared neuropsychological outcome in 4–6 year-old children who were treated with ECMO following repair of congenital heart defects to patients not treated with ECMO and healthy controls. General cognitive ability was within the normal range in both patients groups, yet significantly lower in patients treated with ECMO when compared to healthy controls (14). On more specific neuropsychological tasks, the ECMO group scored significantly lower on left-hand motor skill, visual memory, and spatial construction compared to both cardiac controls and healthy children (14). No group differences were found in sustained attention, general verbal ability, verbal memory, right-hand motor skills, or tactile perception (14). Although providing some insight into long-term neuropsychological outcome in these patients, studies with older children are of interest as these patients may “grow into deficit.” This phenomenon has been described in survivors of severe respiratory failure (2), in which early brain damage becomes only functionally evident at a school-age due to brain maturation and increasing demands on cognitive functioning (23, 24).

Both global and specific brain alterations have been described in survivors of congenital heart disease (25–27). In 8–16 year-old children with transposition of the great arteries that were treated with the arterial switch, significant memory impairment and abnormally small hippocampal volumes were found (20). These impairments were similar to those described previously in patients with acute hypoxemic respiratory failure by the same group (10). Although the neurocognitive deficits were found to be independent of surgical repair, no comparison was made between patients treated with and without ECMO (20).

## Pathophysiological Mechanisms Underlying Neurodevelopmental Deficits

Putting these findings together, it becomes apparent that children with complex congenital heart disease and children who survived severe respiratory failure are at risk of neurocognitive deficits later in life. In both groups, irrespective of ECMO treatment, these deficits lead to difficulties in school. This is highly problematic and underscores the need to understand the pathophysiological mechanisms underlying the long-term neurodevelopmental deficits.

Although some studies seem to suggest that patients treated with ECMO have worse outcomes than patients not treated with ECMO, this is not a consistent finding (9, 11, 14). Both global and specific brain alterations have been described in both of these patient groups. In patients with severe respiratory failure, these brain alterations were largely independent of the need for ECMO treatment (13, 15). However, in a study with school-age survivors of AHRF, patients treated with ECMO seemed to have more widespread brain alterations than patients not treated with ECMO (10). The question rises what pathophysiological mechanisms are underlying these long-term neurodevelopmental deficits. Severity of illness and factors associated with critical illness in general have been described to determine outcome following neonatal critical illness (2). In addition, ECMO treatment may further complicate neurodevelopment by affecting cerebral blood flow and subsequently cerebral autoregulation (28). However, a conclusive cause-effect relationship with ECMO has not been established (28). Moreover, despite lower IQ in patients treated with ECMO compared to patients treated without ECMO (11), the degree of neuropsychological deficits (i.e., specific memory and/or attention problems) and the extent to which these interfere with daily life are similar between these two groups (9, 15, 29). Furthermore, in long-term imaging studies conducted by our group, no differences in (subtle) brain abnormalities were found either between ECMO vs. no-ECMO treated patients, nor between patients treated with veno-arterial and venovenous ECMO (15). These findings suggest that disease processes other than ECMO treatment are underlying the long-term neuropsychological deficits in these patients. An explanation for this may be that, while studies have shown major neurologic complications in about 20% of ECMO-treated patients (30), more subtle brain abnormalities that cannot be detected using standard neonatal MRI seem to be present in both ECMO and non-ECMO patients [12, 14, 28]. While major neurologic complications may lead to severe neurocognitive impairments such as generally lower intellectual ability, these more subtle brain injuries seem to be underlying the long-term neurocognitive deficits in the memory and attention domains observed in the survivors at school-age (29). Neuromonitoring before, during and after neonatal ECMO treatment in these patients is therefore of great importance. Future studies are needed in which neuromonitoring data from near-infrared spectroscopy (NIRS), transcranial Doppler, magnetic resonance imaging (MRI) and/or electroencephalogram (EEG) is coupled with neurodevelopmental outcome to see whether early predictors can be identified.

In survivors of perinatal cardiac failure, the effect of ECMO treatment on long-term brain alterations has, to our knowledge, not been studied. Comparing survivors of cardiac failure to survivors of severe respiratory failure shows both similarities in neurodevelopmental outcome, such as memory deficits with hippocampal alterations (10, 20), as well as differences. In survivors of CHD, multiple neurocognitive domains, such as visuospatial skills, executive functioning, attention and memory (17–21), are found to be compromised. Neurodevelopmental deficits therefore seem to be more widespread in these patient groups than in patients with severe respiratory failure. This may be due to the timing of brain injury. Several studies have shown that brain alterations in patients with CHD already exist prenatally, presumably due to lower oxygen tension in utero (31–33). In contrast, patients with severe respiratory failure are generally born at term and only exposed to deleterious conditions that may affect brain development postnatally. Although evidence from studies in patients with severe respiratory failure seem to suggest otherwise, ECMO treatment in patients with CHD may lead to an even increased risk of diffuse or global brain alterations and subsequent neuropsychological deficits due to its potential effect on cerebral autoregulation (28). However, future studies in CHD patients are needed to assess this notion.

Looking at similarities, we know that all patients described in this review are at risk of hypoxia-ischemia. Evidence has shown that white matter, in particular in the periventricular regions, and the hippocampus are particularly susceptible to hypoxic-ischemic insults (2, 34, 35). Using animal models, premyelinating oligodendrocytes (pre-OLs) in cerebral white matter have been found to be selectively targeted by oxidative stress. These cells account for ~90% of the total oligodendroglial population at 28 weeks of gestation and ~50% at term (35). Increased regional susceptibility of the periventricular white matter is suggested to be due to the distribution of these pre-OLs and relative underdevelopment of distal arterial fields to these areas (35, 36). In addition, studies using animal and *in vitro* models have demonstrated that the hippocampus shows more pronounced changes following hypoxia-ischaemia than other brain structures (34). Indeed, critically ill neonates exposed to hypoxic-ischemic injuries, irrespective of gestatational age or underlying diseasse, have been shown to be at increased risk of periventricular white matter abnormalities and hipopcampal damage (2). Since white matter is important for high-speed transmission of neuronal signals between distant brain regions, aberrations in white matter development affects the orchestration of specific cognitive functions (13, 15). Furthermore, hippocampal alterations have shown to lead to significant memory deficits, which has been described as developmental amnesia in this population (37, 38). In line with this, our group previously found a specific negative association between the maximum dose of vasoactive medication received during first admission (measured by the Vasoactive Inotropic Score; VIS) and long-term verbal and visuospatial memory in survivors of neonatal ECMO and/or congenital diaphragmatic hernia. The association was found in both ECMO and non-ECMO treated patients (9). Although currently speculative, receiving high levels of vasoactive medication in the first period of life may be an indirect marker of temporarily (regional) inadequate brain perfusion. As the hippocampus, is particularly vulnerable for hypoperfusion and/or hypoxia, the association between the VIS and memory may be the indirect result of this pathophysiological mechanism. Future research in which dense and detailed data on continuous oxygen saturation and supplemental oxygen supply is combined with neurodevelopmental outcome is needed to gain further insight in this potential cause-effect relationship.

Finding ways to protect the brain against hypoxic-ischemic injury is therefore an important goal for future studies. The use of pharmacological agents that may have neuroprotective effects may be of great value in critically ill infants. Dexmedetomidine, used in particular for sedation in the pediatric ICU population, may have neuroprotective effects against hypoxic-ischemic damage (39). These effects have been suggested to result from an activation of α_2_-adrenergic receptors by dexmedetomidine, which inhibits inflammation following brain ischemia (40). However, these findings are mostly based on animals models and studies in adult populations (39, 40). Future clinical trials that assess the efficacy and safety of dexmedetomidine in critically ill neonates are therefore needed.

## Future Directions to Improve Neurodevelopment After Neonatal Critical Illness

### Neurorehabilitation

Given the complex interplay of factors that are likely to affect the developing brain in critically ill infants, rehabilitation strategies aimed at improving impaired neuropsychological functions are of great interest in these patients. Our group has recently demonstrated short-term gains in verbal working-memory and associated increased FA in the left superior longitudinal fasciculus (SLF) following Cogmed working-memory training (CWMT) in school-age survivors of neonatal ECMO and/or CDH (29). These findings are in line with the effects demonstrated in other clinical and non-clinical groups after CWMT (8, 41–46). One year after CWMT, neuropsychological follow-up showed that gains in visuospatial memory delayed recall persisted long-term (47). Unfortunately, MRI was not performed at this time, hampering our understanding of the neurobiological mechanisms that may be underlying these long-term changes. Nonetheless, as previous findings showed that over 50% of school-age survivors of neonatal ECMO and/or CDH have long-term visuospatial memory deficits (9), improving memory in these children is of great importance.

Ideally, intervention should take place before memory problems have interfered with school performance. In children with very low birth weight, memory improvements have been found 6 months after CWMT at preschool-age (41), suggesting earlier intervention may lead to similar results. However, these results need to be replicated in preschool survivors of neonatal ECMO and/or CDH with long-term visuospatial memory deficits, as well as in other survivors of critical illness such as following complex cardiac anomalies, before any definitive conclusions can be drawn. In future trials, neuropsychological assessment and neuroimaging should be conducted both immediately and 1 year post-intervention.

These studies demonstrate that neurocognition is malleable with CWMT in survivors of neonatal critical illness. However, it is not the (complete) answer to the long-term neuropsychological deficits observed in these children. Results described in this paper demonstrate that multiple neuropsychological domains are affected in both survivors of neonatal severe respiratory failure and complex cardiac anomalies, either treated with or without ECMO. It is therefore essential to conduct an elaborate neuropsychological assessment before initiating CWMT in survivors of neonatal critical illness to determine its clinical utility. Furthermore, if multiple domains are affected in a child, treatment strategies should ideally affect multiple domains as well. A combination of different intervention programs may therefore be of interest. Findings from both experimental and clinical studies have suggested that multimodal training leads to better results compared to a single training program (48, 49). Exercise training in children has been found to affect memory and learning by targeting the hippocampus (50). Combining such a physical program with cognitive training aimed at improving attention or memory, may strengthen the results and be beneficial in survivors of neonatal critical illness. However, these are future perspectives and of little use in today's clinical practice.

Currently, survivors of neonatal critical illness with long-term neuropsychological deficits may have to manage with practical tools to improve school performance and daily life activities. To improve long-term neurocognitive outcome in these children, we recommend that survivors of neonatal critical illness receive information on the practical implications of the deficits they may experience (e.g., difficulty remembering homework that is due tomorrow or appointments with friends), as well as learn about compensatory techniques or external (memory) aids that may be used to improve their activities of daily living [e.g., errorless learning, mental imagery to improve recall, writing important things down and using a schedule book (51)]. Ideally, this information is personalized to the patient's specific neuropsychological impairments and needs. Personalized information on neuropsychological deficits and practical tools can be realized by conducting neuropsychological assessment to evaluate the degree of neuropsychological deficits, as well as by evaluating the degree to which these deficits affect activities of daily living in the patient (51).

**Figure 1:**
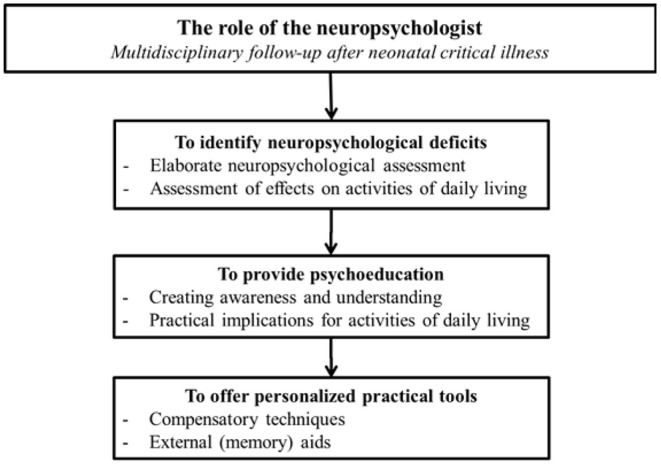
The role of the neuropsychologist in multidisciplinary follow-up after neonatal critical illness.

For all survivors of neonatal critical respiratory and cardiac illness, whether treated with or without ECMO, a long-term follow-up program with regular assessments across development that covers various medical and neurodevelopmental domains is recommended. It is important that follow-up continues until school-age and into adolescence, as these children seem to “grow into deficit” where early brain damage does not become functionally evident until cognitive functioning increases later in life (3). The neuropsychologist as such can play an essential role in (the improvement of) long-term outcome following neonatal critical illness (Figure 1). A multidisciplinary approach to long-term follow-up after neonatal critical illness should therefore be strived for. Please refer to IJsselstijn et al. (52) for more detailed recommendations on multidisciplinary age-appropriate follow-up programs in these patients.

### Prevention

Ideally, the deleterious effects of neonatal critical illness on the neonatal brain should be prevented. This may be (partly) accomplished by fine-tuning therapy or treatment strategies, but may also be achieved with the use of neuroprotective agents in the future. While the specific pathophysiological mechanisms underlying the neurocognitive deficits are yet to be identified, we do know that the hippocampus is highly vulnerable in this population (2). The use of pharmacological agents that may have neuroprotective effects may therefore be of great value in critically ill infants. For instance, maternal allopurinol, which may protect the fetus against hypoxic-ischemic brain injury, is currently being conducted (53). Here, we mention two other potentially neuroprotective agents that are already commonly used in the neonatal intensive care unit (NICU).

Dexmedetomidine, used in particular for sedation in the pediatric ICU population, may have neuroprotective effects on the hippocampus, in particular against hypoxic-ischemic damage (39). These effects have been suggested to result from an activation of α_2_-adrenergic receptors by dexmedetomidine, which inhibits inflammation following brain ischemia (40). As the hippocampus has been found to be vulnerable to both hypoxia-ischemia as well as inflammation (2), this specific mechanisms of action is of interest. However, these findings are mostly based on animals models and studies in adult populations (39, 40). Future clinical trials that assess the efficacy and safety of dexmedetomidine in critically ill neonates are therefore needed that also include neurobiological outcome parameters, such as hippocampal volume. Another agent that may be of interest in this respect is erythropoietin. Erythropoietin is produced by various cell types in the developing brain as a growth factor and as an endogenous neuroprotective response to hypoxia (54). As previously mentioned, in addition to hypoxia, high oxygen concentrations as a result of supplementary oxygen may lead to neonatal brain damage as well (34, 55–58). A recent study in 6-day-old rat pups showed that a single dose of erythropoietin at the onset of hyperoxia (24 h 80% oxygen) improved memory impairment and reduced acute oligodendrocyte degeneration up to the adolescent and adult stage (59). Given the vulnerability of pre-oligodendrocytes in the periventricular white matter during the perinatal period (35), which may potentially be (partly) underlying the attention and memory deficits observed later in life in survivors of neonatal critical illness (9, 11), reducing microstructural abnormalities in these fibers would have direct clinical benefits. In addition, studies have found that erythropoietin may have neurotrophic effects as well by increasing synaptic plasticity in the hippocampus and improving memory formation (59, 60).

The hippocampus shows a high degree of neuroplasticity, which means it has the unique ability to adapt and reorganize in response to internal or external stimuli (58). Although this unfortunately seems to result in more pronounced vulnerability than plasticity—the mechanisms underlying this (im)balance remaining largely unknown—its ability to generate new neurons throughout life does make it a promising target in this respect (58). Trials on potentially neurotrophic agents such as erythropoietin are therefore of interest. In infants with extreme prematurity, hypoxic-ischemic encephalopathy, perinatal stroke, and complex cyanotic heart disease, trials have demonstrated safety, and the potential for efficacy of erythropoietin (61). However, the optimal dose and regimen for neuroprotection in neonates remains largely unknown (62). Future clinical intervention trials assessing the effects of neuroprotective agents before, during or after exposure to both hypoxia and hyperoxia are needed in critically ill neonates.

## Conclusions

Growing up after severe or cardiac respiratory failure in the neonatal period comes with an increased risk of neurocognitive deficits, which seem to be independent of ECMO treatment. In survivors of cardiac failure, widespread neurodevelopmental deficits are more often described than in survivors of respiratory failure, who seem to have more selective neurodevelopmental deficits in attention and memory. This may be due to the timing of brain injury, as brain alterations have been found to exist already *in utero* in patients with congenital heart disease. Nonetheless, academic failure has been reported in both groups. It is therefore of utmost importance that long-term outcome improves in these patient groups. Future studies are needed to identify early biomarkers or risk factors to improve early identification and intervention of children at risk. Furthermore, neuroprotective strategies should be explored. To improve outcome following neonatal critical illness in current practice, psychoeducation, compensatory techniques and external (aids) should become a standard part of (long-term) care following neonatal critical illness.

## Author Contributions

RS designed the manuscript and wrote the first versions while DT agreed upon the final version of the manuscript and provided editorial support.

### Conflict of Interest

The authors declare that the research was conducted in the absence of any commercial or financial relationships that could be construed as a potential conflict of interest.
